# Clostripain, the Missing Link in the Enzyme Blend for Efficient Human Islet Isolation

**DOI:** 10.1097/TXD.0000000000000528

**Published:** 2015-06-24

**Authors:** Magnus Ståhle, Aksel Foss, Bengt Gustafsson, Marko Lempinen, Torbjörn Lundgren, Ehab Rafael, Gunnar Tufveson, Olle Korsgren, Andrew Friberg

**Affiliations:** ^1^ Department of Immunology, Genetics and Pathology, Uppsala University, Uppsala, Sweden.; ^2^ Oslo University Hospital, Rikshospitalet, Oslo, Norway.; ^3^ Department of Transplantation, University Hospital, Gothenburg, Sweden.; ^4^ Department of Transplantation and Liver Surgery, Helsinki University Hospital, Helsinki, Finland.; ^5^ Division of Transplantation Surgery, CLINTEC, Karolinska Institute, Stockholm, Sweden.; ^6^ Transplantation Unit, Department of Surgery, Skåne University Hospital, Malmö, Sweden.; ^7^ Division of Transplantation Surgery, Department of Surgical Sciences, Uppsala University Hospital, Uppsala, Sweden.

## Abstract

Effective digestive enzymes are crucial for successful islet isolation. Supplemental proteases are essential as they synergize with collagenase for effective pancreas digestion. The presence of tryptic-like activity has been implicated in efficient enzyme blends and the present study aimed to evaluate if addition of clostripain, an enzyme with tryptic-like activity, could improve efficacy of the islet isolation procedure.

**Methods:**

Clostripain was added to the enzyme blend just before pancreas perfusion. Islets were isolated per standard method and numerous isolation parameters, islet quality control, and the number of isolations fulfilling standard transplantation criteria were evaluated. Two control organs per clostripain organ were chosen by blindly matching against body mass index, cold ischemia time, hemoglobin A1c, donor sex, and donor age.

**Results:**

There were no differences in pancreas weight, dissection time, digestion time, harvest time, percent digested pancreas, or total pellet volume before islet purification between control or clostripain pancreases. Glucose-stimulated insulin release results were similar between groups. Total isolation islet equivalents, purified tissue volume and islet equivalents/g pancreas as well as fulfillment of transplantation criteria favored clostripain processed pancreases.

**Conclusions:**

The addition of clostripain to the enzyme blend soundly improved islet yields and transplantation rates. It gently aided pancreas digestion and maintained proper islet functionality. The addition of clostripain to the enzyme blend has now been implemented into standard isolation protocols at the isolation centers in Uppsala and in Oslo.

## Abstract 2

Supplemental digital content is available in the text.

Islet transplantation constitutes an efficient treatment option for type 1 diabetes mellitus patients suffering from life-threatening episodes of hypoglycemia and has the benefits of low frequency of serious complications when compared with whole pancreas transplantation.^1-4^ However, the number of organs needed to achieve this effect is higher for islet transplantation,^2,5,6^ considering that all organs accepted for islet processing may not result in a sufficiently large number of well-functioning islets to meet requirements for clinical transplantation. To improve the rate of successful isolations, there have been numerous reports attempting to identify factors of effective digestive enzymes and their application to improve isolation outcome.^7-25^

Supplemental proteases are an essential component for proper release of islets from pancreas parenchyma, they synergize with collagenase for effective pancreas digestion.^8,12,14-16,18,24,26,27^ In our hands, collagenase batches containing a higher level of tryptic-like activity (TLA) were more efficient compared to batches with reduced TLA activity. The seemingly uncontrolled variation of TLA contamination between different batches of collagenases has confounded the islet isolation field with lot-to-lot uncertainty concerning enzyme stability and pancreas digestion efficacy for some time.^7,9,20,28,29^ The TLA was regarded as a typical by-product/contaminant of the collagenase manufacturing process from native *Clostridium histolyticum* bacteria.^12,28^ However, improved purification methods have yielded very pure, intact collagenase products with well-defined Col G (class I) and Col H (class II) fractions.^22,30^

Clostripain is thought to be responsible for TLA in impure collagenase batches, and it has recently become commercially available as a separate purified enzyme. When tried in an animal setting, it was found to have a synergistic effect with neutral protease when used for rat islet isolation.^31^ The aim of the present study was to evaluate if addition of clostripain to the enzyme blend for human pancreas digestion could enhance reproducibility and efficacy of the islet isolation procedure.

## MATERIALS AND METHODS

### Organ Procurement

All participating centers within the Nordic Network for Clinical Islet Transplantation used standard organ procurement procedures.^32^ Centers in the Nordic Network for Clinical Islet Transplantation include in Sweden the University Hospital, Gothenburg; University Hospital, Malmö; Karolinska Institute, Stockholm; and Uppsala University Hospital, Uppsala; in Finland the Surgical Hospital, Helsinki University, Helsinki, Finland; in Norway Oslo University Hospital, Rikshospitalet, Oslo, Norway; and in Denmark Rigshospitalet, Copenhagen, Denmark.

### Donor, Transport, Islet Isolation, and Islet Maintenance

Criteria for donor selection were the same as used for clinical kidney donation with donors between 25 and 69 years of age. Exclusion criteria included a glycosylated hemoglobin A1c higher than 48 mmol/mol according to the International Federation of Clinical Chemistry and Laboratory Medicine.^33^

Details for islet isolation have previously been described.^34,35^ Briefly, methods included intraductal pancreas perfusion with proteolytic enzymes (Vitacyte, Indianapolis, IN) (Serva, Heidelberg, Germany) (Roche, Indianapolis, IN), automated digestion at 37 °C followed by continuous Ficoll (Biochrom, Berlin, Germany)/UW (Viaspan, Apoteket, Uppsala, Sweden) density gradient islet purification from exocrine tissue. Purified tissue was maintained in Connaught Medical Research Labs 1066 media (Corning, Manassas, VA) supplemented with 10 mM HEPES (Invitrogen AB, Stockholm, Sweden), 10 mM nicotinamide (Swedish Pharmacy, Umeå, Sweden), 2 mM l-glutamine (Invitrogen), 50 μg/mL gentamicin (Invitrogen), 5 mM sodium pyruvate (Swedish Pharmacy, Umeå, Sweden), 20 μg/mL ciprofloxacin (Bayer, Leverkusen, Germany), and approximately 10% human serum (Uppsala Blood Bank, Uppsala, Sweden) in a humidified 5% CO_2_ atmosphere at 37 °C overnight. Thereafter, the tissue was kept at 25 °C. Media was changed at day 1, after 21.3 hours (range, 16.8-32.6) of storage for control and after 20.0 hours (range, 17.0-34.1) of storage for clostripain, and every second day thereafter for 1 to 5 days until eventual transplantation. Preparations in the clostripain group were performed between May 2014 and February 2015, whereas isolations in the control group were performed between April 2009 and September 2014.

Before establishing final dosage of enzymes, different concentrations and proportions were evaluated. The final dosages used for isolations in the clostripain group were as follows for the first 2 isolations: 1 vial collagenase (2187 WünchU, 44 Nα-benzoyl-l-arginineethylester hydrochloride [BAEE] U, 1.9% TLA, TLA defined as BAEE U/Wünch U × 100%^12^), 1/2 vial thermolysin (1.49 million neutral protease U^29^), and 1/3 vial clostripain (141 BAEE U, nonactivated), final dosed TLA of 8%. For the following 10 isolations in the clostripain group enzyme dosage were as follows: 1 vial collagenase (2243 WünchU, 44 Nα-benzoyl-l-arginineethylester hydrochloride [BAEE] U, 1.9% TLA, TLA defined as BAEE U/Wünch U × 100%^12^), 1/2 vial thermolysin (1.49 million neutral protease U^29^) and 1/3 vial clostripain (141 BAEE U, non-activated), final dosed TLA of 8%.

For the control group several different collagenases and neutral proteases from different batches were used (**Table S1, SDC**, http://links.lww.com/TP/B177).

This study evaluated one batch of clostripain with an endotoxin level of 191.1 EU/mg. Clostripain, thermolysin, and collagenase were obtained from Vitacyte, and all enzyme components were produced according to the same regulations.

Since this is a retrospective study, two control organs per clostripain organ were chosen by blindly matching against factors possibly impacting isolation outcome. Matching factors are body mass index, cold ischemia time, hemoglobin A1c, donor gender and donor age.

Other parameters of interest are also reported, including organ transport and isolation parameters; preservation media, trimmed pancreas weight, dissection time, pancreas digestion time, harvest time, total digested tissue pellet volume, percent digested pancreas, percent recovery after overnight storage, percent islet purity, islet size distribution, total islet equivalents (IE), and IE/g pancreas.

### Islet Quality Control

Functional islet viability was performed according to standard protocols. The day after islet isolation, quality control assays included glucose-stimulated insulin secretion (GSIS) of 20 handpicked islets using dynamic glucose perifusion (Brandel, London, UK) to calculate the stimulation index (SI) (average of high glucose phase divided with average of low glucose phase).^36^ All insulin values were measured using a human insulin-specific ELISA (Mercordia, Uppsala, Sweden).

### Study Endpoints

The primary endpoint is transplantation rate, that is, the number of human islet isolations fulfilling standard transplantation criteria of greater than 250,000 IE, a GSIS greater than 2.0, and packed tissue volume less than 15 ml at day 1 after isolation.

### Statistical Analysis

Prism 6.0c (GraphPad, La Jolla, CA) was used to perform statistical analysis. The Mann–Whitney *U* test for unpaired groups was used for comparison between experimental groups, and Fisher exact test was used for fulfillment of transplantation criteria rate analysis. Data are presented as mean with range unless specified otherwise. *P* values less than 0.05 were considered significant, whereas *P* values greater than 0.05 were considered nonsignificant. The cumulative sum chart test (Figure 4) is a powerful statistical tool to detect changes in the outcome of the islet isolations in an otherwise standardized procedure of islet isolation.

## RESULTS

### Donor Matching

The data set contains 12 organs isolated using clostripain and 24 matched controls. Because matching was used, there were, consequently, no differences between the groups based on donor factors (Table 1).

**TABLE 1:**
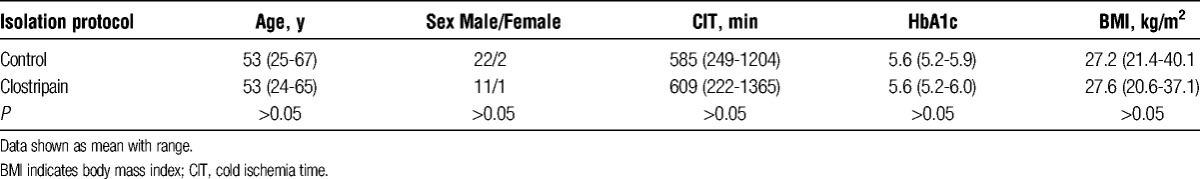
Donor matching parameters

### Isolation Characteristics

There were no differences in terms of collagenase activity between control and clostripain group. There was however a higher amount of thermolysin used in the control group 2,999,333U (range 830,000-3,974,000) compared to clostripain 1,502,500 (range, 1,485,000-1,506,000; *P* = 0.0030).

**FIGURE 1:**
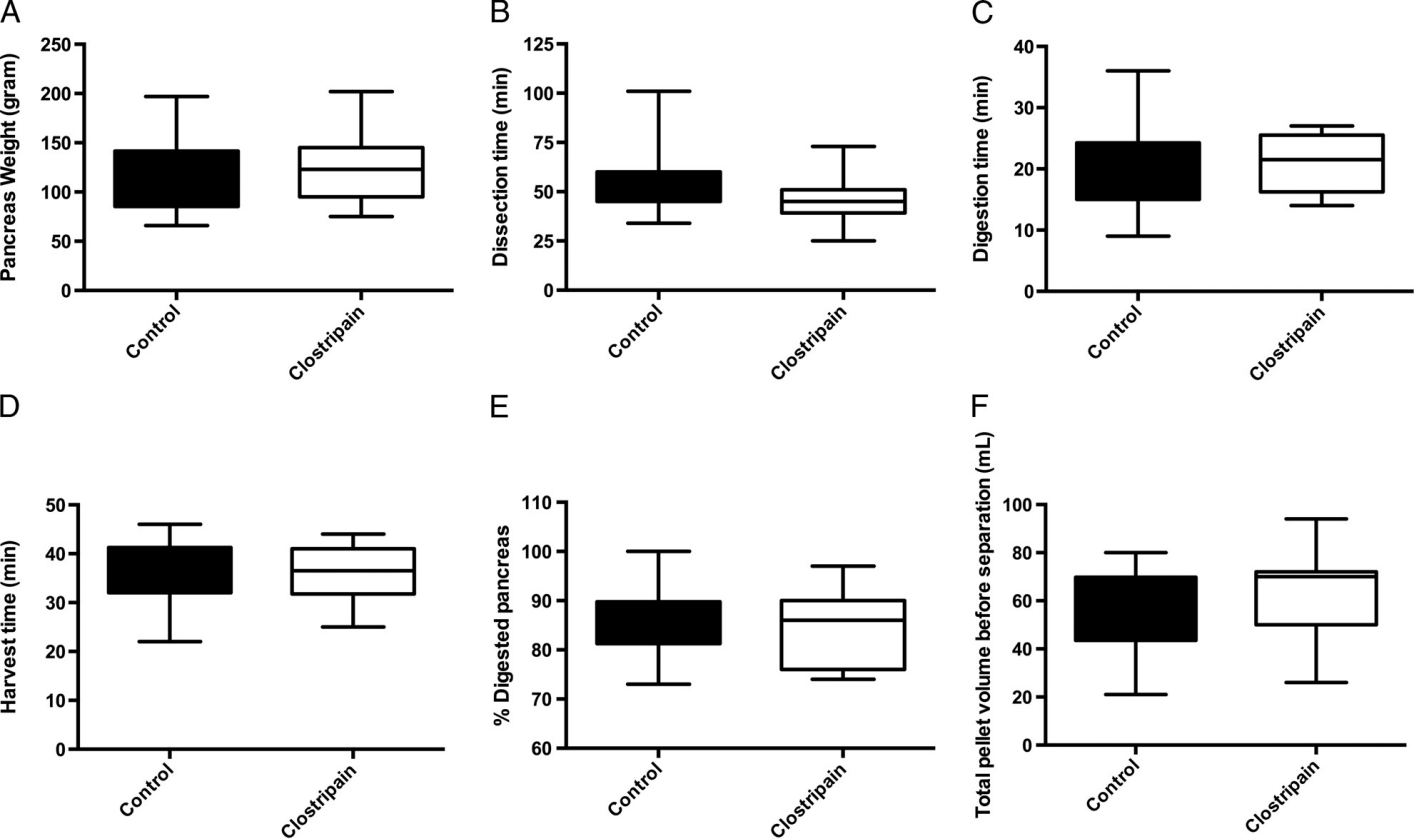
A, Pancreas weight after dissection. B, Dissection time in minutes. C, Digestion time in minutes. D, Harvest time in minutes. E, Percent digested pancreas. F, Total pellet volume before separation.

There were no differences in terms of pancreas weight (Figure 1A), dissection time (Figure 1B), digestion time (Figure 1C), harvest time (Figure 1D), percent digested pancreas (Figure 1E), or total pellet volume before islet purification (Figure 1F) between control pancreases compared with pancreases processed with clostripain.

### Isolation Results

**FIGURE 2:**
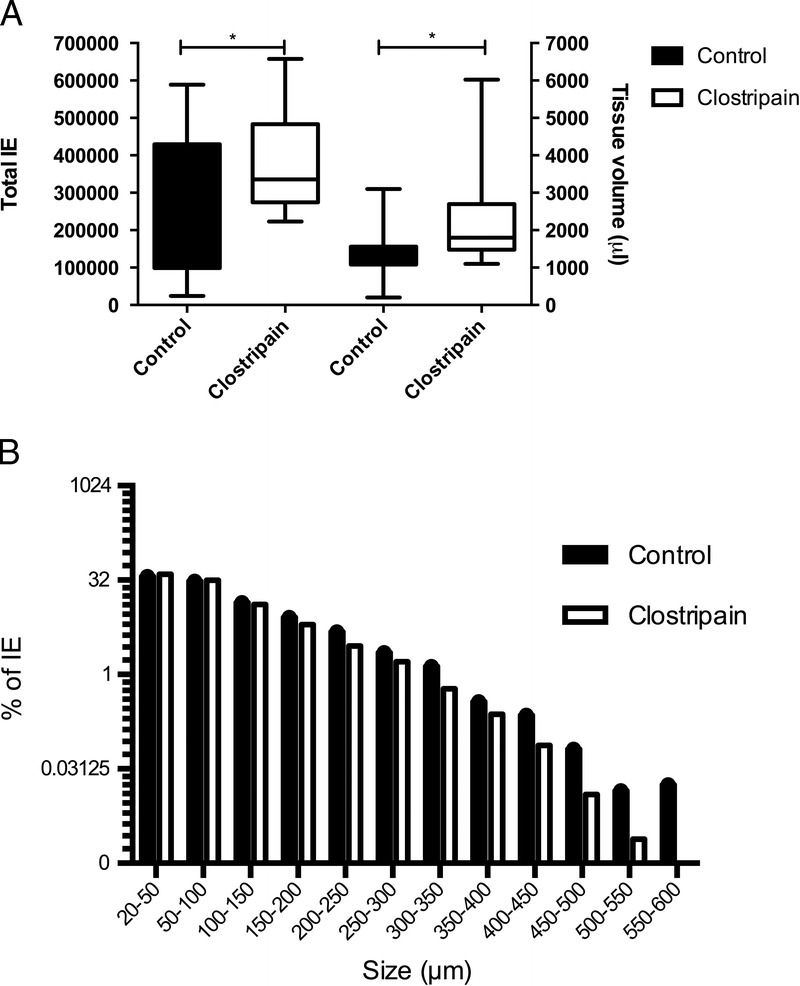
A, Total IE and total purified islet volume at day of isolation. *Significant difference (*P* < 0.05). B, Islet size distribution as percent of total IE number.

There were significant differences between control and clostripain groups, both in terms of IE (254,765 [range 24,130-588,696] vs 391,565 [range 223,368-657,609], respectively, *P* = 0.0199), purified tissue volume (1333 μL [200-3100 μL] vs 2235 μL [1100-6025 μl], respectively, *P* = 0.0090) (Figure 2A), and IE/g pancreas (2498 [233-6086] vs 3598 [range, 2440-6246], *P* = 0.0362, Table 2). For the other isolation outcome parameters, total purity and recovery after 1 day of storage, there were no differences between control and clostripain pancreases (Table 2). Despite use of an additional protease, there was no difference in islet size distribution between the groups (Figure 2B).

**TABLE 2:**
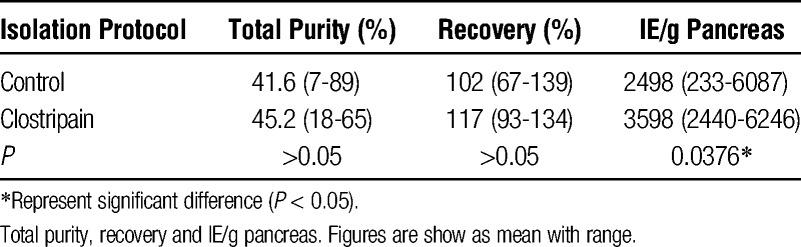
Outcome of Isolations

Similar results were obtained on variables from day 1. After media change, differences remained between control and clostripain groups both in terms of IE (250,812 [range, 24,022-611,739] vs 437,799 [range, 251,304-740,653], *P* = 0.0043), purified tissue volume and IE/g pancreas (*P* < 0.05, data not shown), whereas there were no differences in other outcome parameters, including percent islet recovery and purity.

**FIGURE 3:**
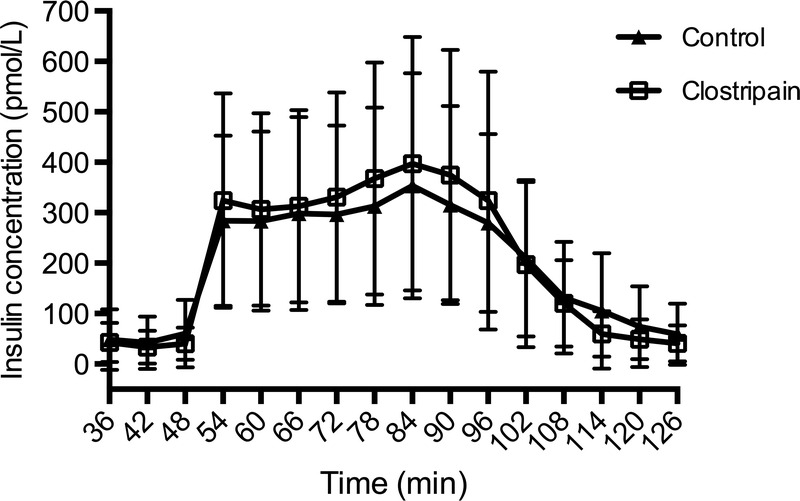
Insulin release curve as response to dynamic glucose perifusion. Islets were perifused with high glucose (16.7 mM) from 42-84 minutes whereas other fractions were exposed to low glucose (1.67 mM).

Quality assessment of the islets revealed no difference between control and clostripain pancreases regarding GSIS SI values (8.0 [range, 1.2-28.6] vs 10.6 [range, 2.0-16.4], respectively, *P* > 0.05) or levels of insulin release (Figure 3). The GSIS perifusion curves show proper biphasic insulin release with an initial spike followed by continued and sustained insulin release until return to low glucose levels.

There was a significant difference when comparing preparations fulfilling transplantation release criteria where 11 of 24 control preparations fulfilled the criteria compared to 12 of 12 in the clostripain group (*P* = 0.0022).

## DISCUSSION

In combination with thermolysin and purified collagenase blends, supplementation with clostripain for the digestion of the human pancreas played a dramatic role in the ability to consistently attain sufficiently high islet yields enabling clinical islet transplantation. In our hands, clostripain gently aids digestion kinetics for consistent release of a high number of intact and fully functional islets from native pancreatic tissues.

Clostripain is an arginine-specific endopeptidase produced and released extracellularly in large amounts as an inactive precursor by *Clostridium histolyticum*. Through autocatalytic removal of a propeptide and a linker peptide, the mature heterodimeric enzyme is formed consisting of a 38-kDa and a 15-kDa chain.^37^ The enzyme is activated by reducing agents and Ca^2+^ ions and shows restricted substrate specificity different to that of trypsin.^38,39^ Addition of clostripain in the enzyme blend for digestion of collagen in human skin biopsies was shown to shorten digestion times, enhance the digestion efficiency as well as to increase the number of cells liberated from the biopsies and their expansion in vitro. This allowed for production of “dermal equivalents based on autologous fibroblasts in clinically acceptable time frame with sufficient repair and regenerative capacities”.^40^

Clostripain is one of the latest contributions to commercially available enzymes. Other available proteases, for example, thermolysin, neutral protease and *Bacillus polymyxa* protease (dispase), all have similar and highly conserved catalytic domains as well as substantial sequence homology. Clostripain differs from these other proteases in that it is a cysteine protease, not a metalloprotease, theoretically capable of activating native pancreatic proelastase to elastase.^41^ In effect, the addition of clostripain may provide not just 1 but 2 additional proteolytic activities to aid efficient release of islets, complementary to other available proteases. Addition of multiple proteases likely complements the enzyme blend more completely than addition of only 1 supplemental enzyme. The relative importance of clostripain in the digestion of a pancreas may vary between different donors, that is, digestion of the pancreas from some donors may be almost complete with addition of only thermolysin or NP, whereas in other donors, the addition of clostripain may be essential for an efficient digestion. This assumed variation in enzymatic digestion requirements between pancreases likely explains the observed consistent high yield islet isolation outcomes using the here described more complete enzyme blend.

One of the challenges in evaluating IE number is that the standard method, used by most islet preparation centers, is subjective and highly dependent on an experienced investigator, resulting in a percent coefficient of variation of 44%. A validated digital image analysis technique was used in the present study to avoid bias, investigator variations as well as overestimation of the islet number and purity.^42,43^

The quality of islets remained good and stable with no observable negative effects on day-of-isolation islet size distribution or day one recovery of IE or purity. One limitation of this study is that we did not obtain islet IE and percentage embedded islet prepurification during standard clinical islet isolations, therefore making it difficult to evaluate if the differences between the groups in terms of obtained IE could be a result of less embedded islets in the clostripain group.

**FIGURE 4:**
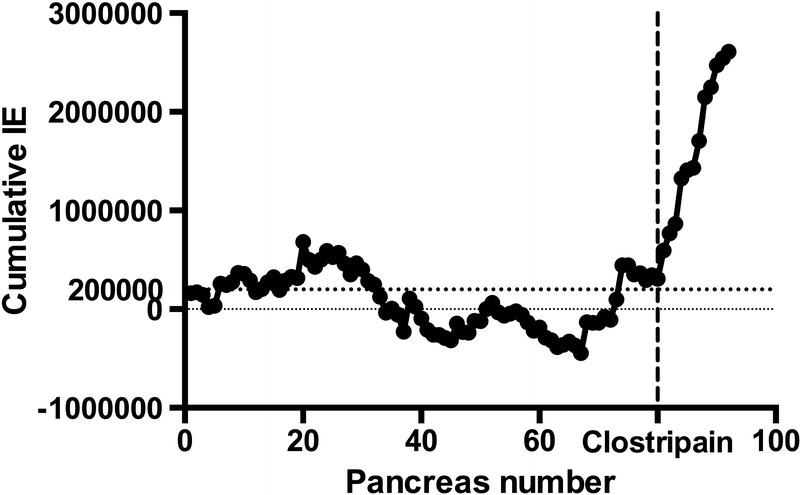
The cumulative IE from a baseline 200,000 IE isolation. For every islet isolation the difference from 200,000 IE is plotted, that is, if the number of IE is above 200,000 IE then the next plot point is a positive addition to the graph, negative if below. To the left of the vertical dashed line are the cumulative IE plot points of 80 standard processed organs with the aim for clinical use. To the right of the vertical dashed line, indicating use of clostripain, are only organs isolated with the specified doses of collagenase, thermolysin and clostripain.

Similarly, the GSIS perifusion SI values were all within good margin for acceptance for clinical use. Considerations for reported results include that islet isolations typically were completed in less than 4 hours and postisolation preservation of islets was in media with approximately 10% human serum which contains antiproteases, for example, α-1-antitrypsin, factors which may limit the exposure of free tissues to harmful levels of damaging enzyme activities.^35,44-46^ Also, the absence of an activating step for clostripain, that is, no exposure to reducing conditions, likely kept clostripain activities low, lower than a fully activated clostripain with an effective TLA of 45% according to the manufacturer's certificate of analysis. Because there were no observed detrimental effects on functional viability, this protocol is now in standard use at our center in Uppsala, Sweden, as well as in our sister-laboratory within the Nordic Network for Clinical Islet Transplantation in Oslo, Norway (personal communication with Hanne Schultz, head of Oslo Isolation Laboratory). Indeed, when comparing the results of non-clostripain supplemented isolations (n = 80) to those with clostripain a pronounced positive trend in IE yields appears (Figure 4).

In comparison to islet isolations without addition of clostripain to the enzyme blend, the rate of transplantations in islet isolations using clostripain increased markedly. The increased rate of transplantation itself should translate to a substantial improvement in the cost benefits of islet transplantation from an insurance and health care perspective.^47,48^

The removal of TLA activity from collagenase products benefits islet isolation by reducing degradation of the collagenase and by allowing controlled supplementary dosing of proteases and clostripain. Our previous study demonstrated improved isolation outcomes with batches containing a contamination of TLAs of 5.6% to 12%,^12^ Addition of clostripain to achieve a TLA level up to 8% used herein was in good accordance with this previous report. However, the markedly increased transplantation rate also indicates an improved collagenase activity possibly achieved by removal of the contaminating TLA activity in the collagenase products and thereby unwanted enzyme degradation (Figure 5).

## Supplementary Material

SUPPLEMENTARY MATERIAL
